# Domestic Economy in Hospitals

**Published:** 1894-11-17

**Authors:** 


### Editor's Letter-Box.

[Our correspondents are reminded that proxility is a great bar to publication, and that brevity of style and conciseness of statement greatly facilitate early insertion.]

### DOMESTIC ECONOMY IN HOSPITALS.

A Reply to Mr. Ryan.

Our correspondent writes: Every workman is unhappily liable to make a slip with his tools, but his concern is far greater should he inadvertently wound others as well as himself. Mr. Ryan is justly indignant at the obvious oversight in the article of October 13th, in which the cost of butter at St. Mary's (where patients provide nothing) is unfavourably contrasted with the cost of the same article at University and Middlesex, where it is provided by the patients themselves. Allow me to point out, however, that Mr. Ryan is entirely beside the mark in his subsequent comments. If he looks through the article in question again he will find that no comparison whatever is instituted, as he seems to suppose, between the general cost of provisions at St. Mary's and Middlesex, so that his justification, however interesting and instructive, is simply brought in *apropos de bottes*. The article alluded to the extraordinary difference in the cost of provisions per bed in the various metropolitan hospitals, as evidenced by the figures of £32 at St. Mary's and £15 at University. It is not unreasonable to suppose that the authorities of these hospitals share the astonishment of the public at the way in which these figures work out, although in the absence of anything like a common standard of expenditure, and of sufficient data concerning household management, it is quite impossible to ascertain whether the one side should be accused of parsimony, or the other of extravagance, or whether, indeed, by due interpretation of the figures, the cost of provisions at both hospitals may not be shown to be identical. At all events, the authorities are assumed (is it too unnatural a supposition ?) to take advantage of the uniform system of accounts, and compare expenses item by item. The butter (with a renewed *mea culpa*) drops out of the question. The eggs remain. Perceiving that St. Mary's spent in 1893 an average of £2 2s. 9d. on this article, against an average of £1 2s. 8d. at University, the authorities are assumed to continue the enquiry by comparing the cost of the same article at other hospitals. They turn to Middlesex, a hospital with an average for provisions not greatly below their own, and with exceptional food expenses owing to its chronic wards. They find that here again the average spent on this item is considerably below their own (£1 16s. per occupied bed), and it is assumed that they will then proceed to ascertain the exact cause of the difference. No attack upon St. Mary's was intended by this brief indication of the lines on which the uniform system of accounts may lead to satisfactory results. They are the lines of common sense and practical domestic economy, and only by this system of continual mutual comparison and checking of expenditure can the greatly-to-be-desired common standard of working expenditure be evolved.

